# Triglyceride Form of Docosahexaenoic Acid Mediates Neuroprotection in Experimental Parkinsonism

**DOI:** 10.3389/fnins.2018.00604

**Published:** 2018-08-28

**Authors:** Maricel Gómez-Soler, Begoña Cordobilla, Xavier Morató, Víctor Fernández-Dueñas, Joan C. Domingo, Francisco Ciruela

**Affiliations:** ^1^Unitat de Farmacologia, Departament de Patologia i Terapèutica Experimental, Facultat de Medicina, Institut d’Investigació Biomèdica de Bellvitge, Universitat de Barcelona, Barcelona, Spain; ^2^Institut de Neurociències, Universitat de Barcelona, Barcelona, Spain; ^3^Departament de Bioquímica i Biologia Molecular, Facultat de Biologia, Universitat de Barcelona, Barcelona, Spain

**Keywords:** docosahexaenoic acid, Parkinson’s disease, beam walking test, motor coordination, balance, omega-3 polyunsaturated fatty acids

## Abstract

Parkinson’s disease (PD) is a neurodegenerative disorder of unknown etiology. The main treatment of PD consists of medication with dopamine-based drugs, which palliate the symptoms but may produce adverse effects after chronic administration. Accordingly, there is a need to develop novel neuroprotective therapies. Several studies suggest that omega-3 polyunsaturated fatty acids (*n*-3 PUFA) might provide protection against brain damage. Here, we studied several experimental models of PD, using striatal neuronal cultures, striatal slices, and mice, to assess the neuroprotective effects of docosahexaenoic acid (DHA), the main *n*-3 PUFA in the brain, administered in its triglyceride form (TG-DHA). Hence, we determined the beneficial effects of TG-DHA on neural viability following 6-hydroxydopamine (6-OHDA)-induced neurotoxicity, a well-established PD model. We also implemented a novel mouse behavioral test, the beam walking test, to finely assess mouse motor skills following dopaminergic denervation. This test showed potential as a useful behavioral tool to assess novel PD treatments. Our results indicated that TG-DHA-mediated neuroprotection was independent of the net incorporation of PUFA into the striatum, thus suggesting a tight control of brain lipid homeostasis both in normal and pathological conditions.

## Introduction

Parkinson’s disease (PD) is the second most common neurodegenerative disorder after Alzheimer’s disease, and affects approximately 1% of individuals over the age of 60 (de Lau and Breteler, 2006). The main clinical features of PD include asymmetric onset of bradykinesia (slowness to initiate voluntary movements with progressive reduction in speed and amplitude of repetitive actions), rigidity, resting tremor, and posture instability (Meissner et al., 2011). All these symptoms may appear after the selective death of approximately 60% of striatal dopaminergic afferent fibers arising from the substantia nigra pars compacta (Braak and Del Tredici, 2008). The principal therapeutic approach to PD management since the 1970s has been to restore striatal levels of dopamine by means of the precursor L-3,4-dihydroxyphenylalanine (L-DOPA) or other dopaminergic agonists (Poewe, 2009). However, this kind of treatment is only palliative, and chronic consumption of dopamine-based drugs may also induce a number of adverse effects (i.e., L-DOPA induced dyskinesia) (Huot et al., 2013). Consequently, research has also focused on going beyond symptomatic treatments in the quest for neuroprotective agents that may impede or delay disease progression.

The etiology of PD is not fully known. Although genetic and environmental factors have both been described as possible causes for PD, a complete explanation of the pathogenesis of this disorder is still lacking (Puschmann, 2013; Ascherio and Schwarzschild, 2016). Nevertheless, it is well established that some of the mechanisms that lead to PD include oxidative stress, mitochondrial dysfunction, and/or inflammation (Schapira, 2008; Laye, 2010; Blesa et al., 2015). Accordingly, a number of agents including caffeine, nicotine, coenzyme Q10, and omega-3 polyunsaturated fatty acids (*n*-3 PUFA) have been proposed to counteract these processes (Müller et al., 2003; da Silva et al., 2008; Quik et al., 2009; Prediger, 2010; Seidl and Potashkin, 2011). Of these, *n*-3 PUFA represent one of the most challenging molecules, since they are not only natural dietary substances, but they are also essential components of neuronal membranes, and are known to be involved in the inflammatory response to tissue damage. Thus, a potential anti-inflammatory role for *n*-3 PUFA has been proposed, based on their capacity to inhibit brain cyclooxygenase or to improve mitochondrial respiratory function (Massaro et al., 2006; Stanley et al., 2012). In support of this notion, an anti-inflammatory effect of *n*-3 PUFA supplementation in older individuals has been demonstrated; for instance, administration of fish oil-based lipid emulsions was observed to increase circulating levels of anti-inflammatory cytokines (Barros et al., 2013; Berger et al., 2013).

In addition, epidemiological and pre-clinical data alike point to neuroprotective effects of *n*-3 PUFA in neurodegenerative disorders. The most abundant *n*-3 PUFA in the brain, docosahexaenoic acid (DHA), is also one of the most extensively studied (Dyall, 2015), and several studies conducted in PD animal models have reported the beneficial effects of DHA. For example, DHA administration reduced L-DOPA-induced dyskinesia in parkinsonian monkeys (Wurtman et al., 2006), and DHA dietary supplementation reduced apoptosis of dopaminergic cells in the brain of MPTP-treated mice (Bousquet et al., 2008). Besides pre-clinical data, there is also some information on the use of DHA in humans, although large, well-designed intervention studies on the effects of DHA in normal aging and in neurodegenerative disorders are still lacking (Dyall, 2015). Nevertheless, a recent clinical trial studied the role of DHA in reducing dyskinesia in PD (ClinicalTrials.gov identifier: NCT01563913), while in another study it was found that DHA supplementation correlated well with a lower risk of depression associated with PD (da Silva et al., 2008). Further data in the literature also support a role for DHA supplementation in the management of depression and cognitive impairments. Thus, several studies have reported that intake of dietary *n*-3 PUFA is associated with a lower risk of depression (for review see Grosso et al., 2016), and similarly, that there is an inverse correlation between *n*-3 PUFA intake and age-related cognitive decline (for review see Pusceddu et al., 2016).

Overall, DHA and other natural dietary substances show potential as adjunctive therapies for PD, since they may exert neuroprotective effects. Here, we aimed to provide a pre-clinical paradigm by using the well-known 6-hydroxydopamine (6-OHDA)-PD model together with a novel behavioral test (beam walking test), which may serve to assess the potential of these molecules to improve PD treatment.

## Materials and Methods

### Reagents

Triglyceride of docosahexaenoic acid (TG-DHA) obtained by enzymatic synthesis was kindly supplied by Brudy Technology (Barcelona, Spain). This oil contains more than 70% TG-DHA and more than 90% of ω-3 of total fatty acids (Mancera et al., 2017). The stock TG-DHA solution in 100% ethanol was diluted with the indicated aqueous buffer before use. All other compounds were obtained from external sources: 6-hydroxydopamine (6-OHDA) (Sigma-Aldrich, St. Louis, MO, United States) and 3-(4,5-dimethylthiazol-2-yl)-2,5-diphenyltetrazolium bromide tetrazolium (MTT) (Sigma-Aldrich). The primary antibodies used were rabbit anti-TH polyclonal antibody (Millipore, Temecula CA, United States) and rabbit anti-α-actinin-1 polyclonal antibody (Santa Cruz biotechnology, Dallas TX, United States). The secondary antibodies used were horseradish peroxidase (HRP)-conjugated goat anti-rabbit IgG (Pierce Biotechnology, Rockford, IL, United Staes) and Alexa Fluor 488-conjugated donkey anti-rabbit IgG (Jackson ImmunoResearch Laboratories Inc., West Grove, PA, United States).

### Animals

CD-1 wild-type mice (weight 22–27 g), either males or females, from the University of Barcelona animal facility were used. Interestingly, no significant effect of sex was found in the subsequent experiments using animals. The University of Barcelona Committee on Animal Use and Care approved the study protocol. Animals were housed and tested in compliance with the guidelines given in the Guide for the Care and Use of Laboratory Animals (Clark et al., 1997) and following European Union directives (2010/63/EU). Every effort was made to minimize animal suffering and the number of animals used. Animals were housed in groups of five in standard cages with *ad libitum* access to food and water and maintained under controlled standard conditions (12 h dark/light cycle starting at 7:30 am, 22°C temperature, and 66% humidity). Behavioral testing was performed and between 2 pm and 7 pm, in mice aged 2–3 months.

### Striatal Slices and MTT Assay

Mouse brain was removed and placed in ice-cold KREB-saline buffer (124 mM NaCl, 4 mM KCl, 1.5 mM CaCl_2_, 1.5 mM MgSO_4_, 1.25 mM KH_2_PO_4_, 10 mM D-glucose, and 26 mM NaHCO_3_) bubbled with 95% O_2_ up to pH 7.4. Brain slices (300 μm) were rapidly prepared using a Leica VT1200 vibratome (Leica Lasertechnik GmbH, Heidelberg, Germany). The striatum was dissected in KREBs buffer at 4°C and allowed to recover for 30 min in KREBs buffer at 37°C. Subsequently, striatal slices were incubated with the indicated 6-OHDA concentration in the absence or presence of TG-DHA in KREBs buffer at 37°C and toxicity was evaluated by measuring cell uptake of 3-(4,5-dimethylthiazol-2-yl)-2,5-diphenyltetrazolium bromide (MTT), as previously described (Liu et al., 1997). The tetrazolium ring of MTT is cleaved by active dehydrogenases in living cells to produce a precipitated formazan salt that is solubilized by DMSO to give a colored compound. After stirring the mixture for 5 min, optical density was measured at 540 nm in a POLARstar plate reader (BMG Labtech, Durham, NC, United States) to determine cell death rates.

### Striatal Neuronal Cultures

Primary striatal neurons were cultured from E18 CD-1 mouse embryos. Briefly, after dissection, the striatum was treated with 1.25% trypsin (Sigma-Aldrich) for 10 min and mechanically dissociated with a flame-polished Pasteur pipette. Neurons were plated onto poly-D-lysine-coated (0.1 mg/mL) and laminin-coated (0.01 mg/mL) wells at a density of 80,000 cells/cm^2^ on a 96-well plate in minimum essential medium (Invitrogen, Carlsbad, CA, United States) supplemented with 10% horse serum, 10% bovine serum, 1 mM pyruvic acid, and 0.59% glucose. After 4–14 h, the medium was substituted with Neurobasal medium supplemented with penicillin (100 U/mL), streptomycin (100 μg/mL), 0.59% glucose, and B27 supplement (Invitrogen Carlsbad). Neurons were maintained at 5% CO_2_, 37°C, and 95% humidity for 11 days. Subsequently, neurons were treated with increasing concentrations (0, 10, 50, and 100 μM) of TG-DHA growing medium for 3 days. Next, neurons were incubated with 6-OHDA (500 μM) for 24 h. Neuronal death was assessed by MTT assay (see above).

### Surgery

Experimental parkinsonism was induced in mice by means of bilateral striatal lesion as previously described (Fernández-Dueñas et al., 2014). In brief, mice were anesthetized with a ketamine/xylazine combination (75 mg/kg ketamine hydrochloride/10 mg/kg xylazine hydrochloride, intraperitoneally) (Merial Laboratorios S.A. Barcelona, Spain and Laboratorios Calier S.A. Barcelona, Spain, respectively) and immobilized in an adapted digital lab stereotaxic device (Stoelting Co., Wood Dale, IL, United States). Then, an incision (0.5 cm) was performed in the skin of the skull to bilaterally lesion both the right and the left striatum with 6-OHDA (8 μg of 6-OHDA in 4 μl of saline solution containing 0.05% ascorbic acid; Sigma-Aldrich, St Luis, MO, United States). Following the mouse atlas (Franklin and Paxinos, 2008), the stereotaxic coordinates with respect to the bregma were: AP = 1.0 mm, ML = ± 1.7 mm, and DV = -3.5 mm. The 6-OHDA solution was injected manually at a rate of 1 μl/min, and after injection, the needle was left in place for 5 min before retracting it slowly to prevent reflux. Mice were then quickly warmed and returned to their cages. In addition, a similar number of mice were injected with vehicle (saline) and used as a control to discriminate the possible effects of surgery. After surgery, animals (vehicle- and 6-OHDA-lesioned) were treated either with intragastric (i.g.) vehicle (0.5% methylcellulose and 2% DMSO) or TG-DHA (250 mg/kg) for 22 consecutive days (**Figure 2A**). Blood samples were obtained after fasting (8–12 h) by cardiac puncture and centrifuged to separate the plasma fraction from red blood cells (RBCs). The cortex and striatum were dissected, and total membrane extracts obtained as described below. Plasma and membrane extracts from RBCs, cortex, and striatum were immediately frozen and stored at -80°C until used for lipidomic analyses.

### Immunohistochemistry

Mice were fixed and coronal brain sections (50–70 μm) obtained as previously described (Taura et al., 2015). Slices were collected in Walter’s Antifreezing solution (30% glycerol, 30% ethylene glycol in PBS, pH 7.2) and kept at -20°C until processing. On the day of the experiment, slices were washed three times in PBS, permeabilized with 0.3% Triton X-100 in PBS for 2 h and rinsed again three more times with wash solution (0.05% Triton X-100 in PBS). The slices were then incubated with blocking solution (10% NDS in wash solution; Jackson ImmunoResearch Laboratories Inc.) for 2 h at R.T. and subsequently incubated overnight with the primary antibodies at 4°C. After two rinses (10 min each) with 1% NDS in wash solution, sections were incubated for 2 h at R.T. with the appropriate secondary antibodies conjugated with Alexa dyes (Invitrogen, Carlsbad, CA, United States), then washed (10 min each) twice with 1% NDS in wash solution, twice more with PBS, and mounted on slides. Fluorescence striatal images were captured using a Leica TCS 4D confocal scanning laser microscope (Leica Lasertechnik GmbH, Heidelberg, Germany).

### Fatty Acid Analysis

Fatty acid composition was determined using the method described by Lepage and Roy (Lepage and Roy, 1986). Total lipids, containing 0.01% butylhydroxytoluene as antioxidant, were transesterified with acetyl chloride for 60 min at 373 K. Gas chromatography analysis was performed using a Shimadzu GCMS-QP2010 Plus gas chromatograph-mass spectrometer (Shimadzu, Kyoto, Japan). Fatty acid methyl ester peaks were identified by their elution pattern and relative retention times with respect to a reference mixture (GLC-744 Nu-Chek Prep. Inc., Elysian MN, United States). The results were expressed in relative amounts (molar percentage of total fatty acids). For specific fatty acids, the following indexes were calculated as previously described (Cacabelos et al., 2014; de Luis et al., 2016): saturated fatty acids (SFA) = Σmol% of SFA; unsaturated fatty acids (UFA) = Σmol% of unsaturated fatty acids; monounsaturated fatty acids (MUFA) = Σmol% of monoenoic fatty acids; polyunsaturated fatty acids series *n*-3 (PUFA *n*-3) = Σmol% of polyunsaturated fatty acids *n*-3 series; polyunsaturated fatty acids series *n*-6 (PUFA *n*-6) = Σmol% of polyunsaturated fatty acids *n*-6 series; *n*-6/*n*-3 ratio = (Σmol% of PUFA *n*-3)/(Σmol% of PUFA*n*-6); AA/DHA ratio = (mol% (20:4*n*-6)/(mol% of 22:6*n*-3); peroxidizability index (PI) = [(0.025 × Σmol% monoenoic) + (1 × Σmol% dienoic) + (2 × Σmol% trienoic) + (4 × Σmol% tetraenoic) + (6 × Σmol% pentaenoic) + (8 × Σmol% hexaenoic)]; double bond index (DBI) = (Σmol% monoenoic × 1) + (Σmol% dienoic × 2) + (Σmol% trienoic × 3) + (Σmol% tetraenoic × 4) + (Σmol% pentaenoic × 5) + (Σmol% hexaenoic × 6); anti-inflammatory index (AI) = [Σmol% of (20:3*n*-6)+ (20:5*n*-3)+ (22:5*n*-3)+(22:6*n*-3)]/[mol% (20:4*n*-6)].

### Membrane Preparation

Striatal membranes were prepared as previously described (Fernández-Dueñas et al., 2015). In brief, the striatum was dissected and rapidly homogenized in ice-cold 10 mM Tris–HCL (pH 7.4), 1 mM EDTA, and 300 mM KCl buffer with Polytron at setting five for three periods of 10 s each. The homogenate was centrifuged for 10 min at 1000 × *g* and the resulting supernatant centrifuged again for 30 min at 12000 × *g*. The pellet was resuspended in the same buffer, and protein content was determined by means of the Bradford assay (Pierce Biotechnology).

### Gel Electrophoresis and Immunoblotting

Sodium dodecyl sulfate polyacrylamide gel electrophoresis (SDS/PAGE) was performed using 10% polyacrylamide gels. Proteins were transferred to PVDF membranes using a semidry transfer system and immunoblotted with the indicated primary antibody and then HRP-conjugated goat anti-rabbit IgG (1:30000). The immunoreactive bands were visualized using a chemiluminescent detection kit (Pierce Biotechnology) and an Amersham Imager 600 (GE Healthcare Europe GmbH, Barcelona, Spain).

### Beam Walking Test

We assessed the motor coordination and balance of mice by measuring their ability to traverse a narrow beam to reach an enclosed safety platform (Luong et al., 2011). The beam consisted of a long strip of wood (1 m) with a variable width ranging from 28 to 5 mm. The beam was placed in an inclined position, with one end mounted on a 50-cm high narrow support and the other end attached to an enclosed box on a 100-cm high narrow support. During training, mice were placed at the start of the beam and trained over 2 days to traverse the beam to the enclosed box. Once the mice were trained, they performed one trial and the time to traverse the beam was quantified and registered. This test is particularly useful for detecting subtle deficits in motor skills and balance that may not be detected by other motor tests, such as the Rotarod test (Luong et al., 2011).

### Statistics

Data are represented as means ± S.E.M. Comparisons among experimental groups were performed by Student’s *t-*test, one-way ANOVA with Dunnett’s or Tukey’s *post hoc* test, or by two-way ANOVA followed by Bonferroni’s *post hoc* test using GraphPad Prism 6.01 (San Diego, CA, United States), as indicated. Statistical difference was accepted when *P* < 0.05. Linear logistic regression and correlation analysis were performed in selected experiments.

## Results

### TG-DHA Reduced 6-OHDA-Mediated Neurotoxicity *in vitro* and *ex vivo*

**FIGURE 1:**
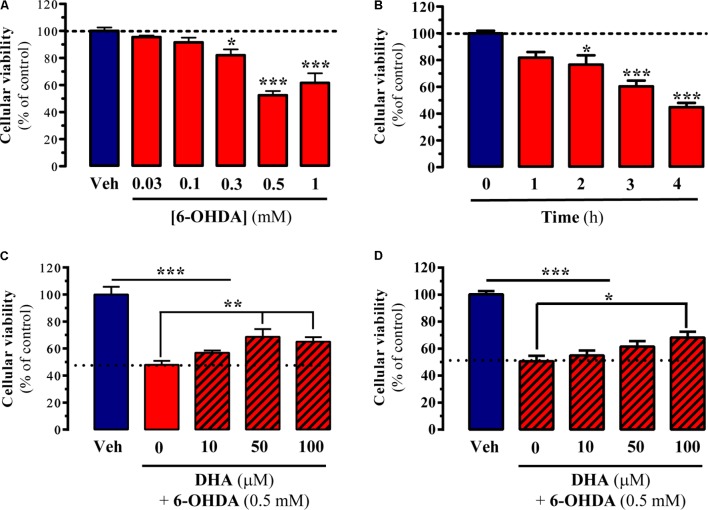
Neuroprotective effect of TG-DHA *ex vivo* following 6-OHDA treatment. **(A)** Striatal slices were treated either with vehicle (Veh) or increasing concentrations of 6-OHDA (0.03-1 mM) for 4 h before neuronal death was determined by MTT assay. **(B)** Striatal slices were treated with 6-OHDA (0.5 mM) for 1, 2, 3, and 4 h before neuronal death was determined by MTT assay. **(C)** Striatal slices were treated with 6-OHDA (0.5 mM) for 4 h in the absence or presence of increasing concentrations of TG-DHA (10–100 μM) before neuronal death was determined by MTT assay. **(D)** Striatal primary neurons were treated with 6-OHDA (0.5 mM) for 24 h in the absence or presence of increasing concentrations of TG-DHA (10–100 μM) before neuronal death was determined by MTT assay. Results are presented as means ± SEM of three independent experiments performed in triplicate. ^∗^*P* < 0.05, ^∗∗^*P* < 0.01, and ^∗∗∗^*P* < 0.001 one-way ANOVA with Dunnett’s *post hoc* test when compared to vehicle treatment **(A,B)** or Tukey’s *post hoc* test for multiple comparisons **(C,D)**.

The main aim of the present study was to evaluate the possible neuroprotective effects of DHA in several experimental models of PD. To this end, in cell, *in vitro*, and *ex vivo* experimental models of parkinsonism were implemented. First, we studied the capacity of TG-DHA to reverse 6-OHDA-induced toxicity in striatal primary cultures and striatal slices. 6-OHDA has been widely used to damage dopaminergic neurons *in vitro* (Ding et al., 2004), thus we expected to obtain valuable information regarding the TG-DHA dosages to employ in subsequent experimental models. We first evaluated cell viability of striatal slices after challenging them with increasing concentrations of 6-OHDA for 4 h. As shown in **Figure 1A**, cell death was dose-dependently induced by 6-OHDA, from which we selected 0.5 mM for further experiments. We also determined the time-course of 6-OHDA-induced *ex vivo* cell death in order to select the most appropriate incubation time. As shown in **Figure 1B**, the maximal effect of 6-OHDA was observed 4 h after incubation. Having established the 6-OHDA dose and incubation time, we next investigated the capacity of TG-DHA to block or attenuate cell death. Accordingly, we incubated slices with 6-OHDA (0.5 mM for 4 h) in the absence or presence of increasing concentrations of TG-DHA and found that TG-DHA dose-dependently promoted cell viability after 6-OHDA treatment, although it did not completely preclude induced cell death (**Figure 1C**). Based on the results obtained in striatal slices, we also evaluated the effects of TG-DHA co-incubation following 6-OHDA treatment in striatal primary cultures. Our results indicated that again, TG-DHA dose-dependently and partially blocked 6-OHDA-induced cell toxicity in striatal primary cultures (**Figure 1D**).

### TG-DHA Counteracted 6-OHDA-Mediated Neurotoxicity in Mice

**FIGURE 2:**
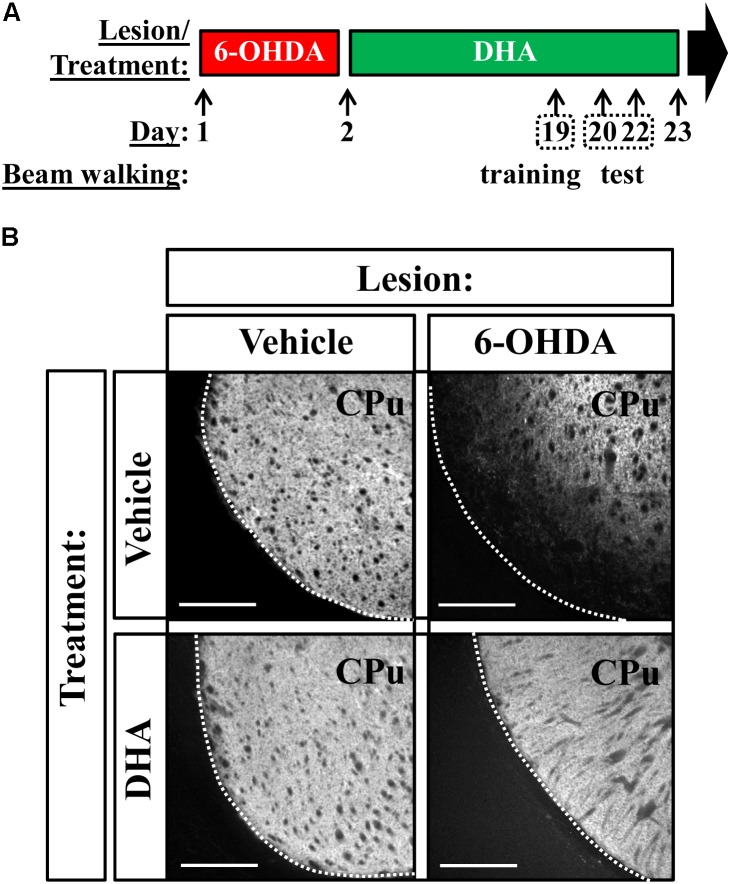
TG-DHA precludes 6-OHDA-mediated reduction in striatal TH. **(A)** Treatment schedule depicting the 6-OHDA lesion and the TG-DHA administration regimen (250 mg/kg, i.g.) and behavioral testing (beam walking test). **(B)** Photomicrographs showing immunohistochemistry detection of TH in the striatum of vehicle- (saline) and 6-OHDA-lesioned mouse treated either with vehicle (saline) or TG-DHA as shown in **(A)**. Scale bar: 350 μm. CPu: caudate putamen.

Having determined the capacity of TG-DHA to partially counteract 6-OHDA-mediated toxicity *in vitro* and *ex vivo*, we studied an animal model of PD. To this end, we bilaterally lesioned mice striatum by means of stereotaxic injection of 6-OHDA, as previously described (Fernández-Dueñas et al., 2014), and treated animals as indicated in **Figure 2A**. When assessing TH distribution in the striatum, which corresponds to existing dopaminergic neurons, we observed a considerable reduction in immunostaining only in 6-OHDA treated animals, while TG-DHA seemed to protect against the death of dopaminergic cells (**Figure 2B**).

**FIGURE 3:**
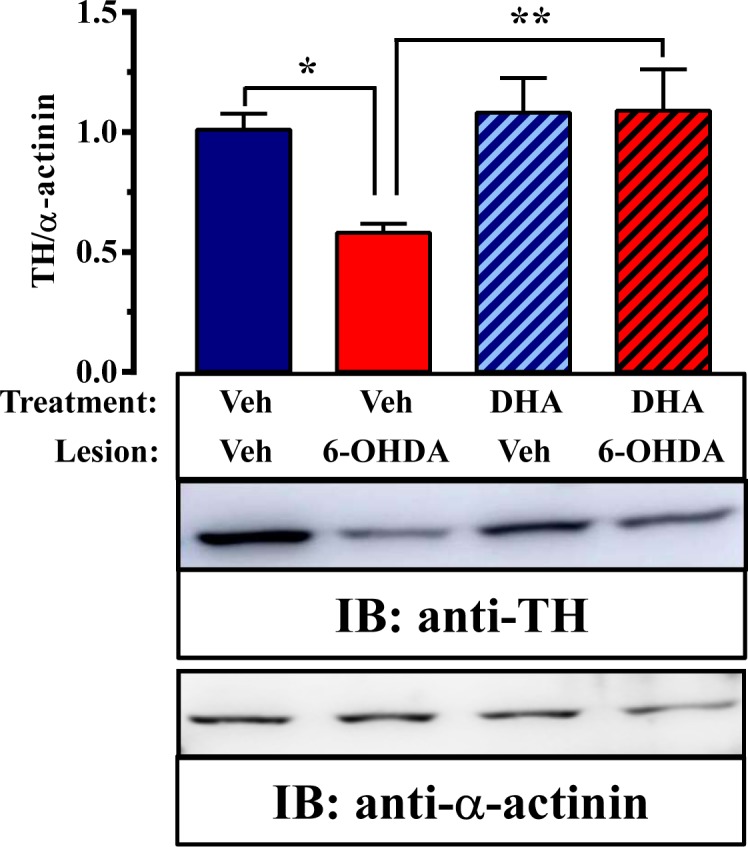
TG-DHA effect on 6-OHDA-mediated neurotoxicity in mice. Representative immunoblot showing TH immunoreactivity in striatal membranes from vehicle- (Veh) and 6-OHDA-lesioned mice treated either with vehicle (Veh) or TG-DHA (see **Figure 2A**). Striatal membranes were analyzed by immunoblot (5 μg of protein/lane) using rabbit anti-TH (1 μg/ml) and rabbit anti-α-actinin (1 μg/ml). The primary bound antibody was detected using a HRP-conjugated goat anti-rabbit antibody (1/30,000). A representative blot of four samples is shown. TH immunoreactive band intensities were measured by densitometric scanning and normalized with respect to the corresponding α-actinin immunoreactive band intensity for each sample. Results are expressed as means ± SEM (n = 12 animals) relative to those for vehicle-treated animals. ^∗^P < 0.05, ^∗∗^P < 0.01, and ^∗∗∗^P < 0.001 two-way ANOVA with Bonferroni’s post hoc test.

Next, we assessed TH expression in the striatum by immunoblot analysis to quantify the extent of the lesion and the possible neuroprotection mediated by TG-DHA (**Figure 3**). Interestingly, while an effect of TG-DHA treatment [*F*_(1,44)_ = 58.4, *P* = 0.0199] was confirmed by two-way ANOVA analysis of the data, no effect of 6-OHDA lesion [*F*_(1,44)_ = 3.1, *P* = 0.0853] or interaction between both factors [*F*_(1,44)_ = 3.349, *P* = 0.074] was observed. In addition, significant differences (*P* < 0.05) in TH expression between vehicle- and 6-OHDA-lesioned mice were confirmed by Bonferroni’s *post hoc* analysis. Accordingly, based on the different TH expression, our results suggested that TG-DHA treatment protects against 6-OHDA-mediated toxicity.

### Effects of TG-DHA Treatment on Fatty Acid Profile in Mouse Striatum

Several studies have hypothesized that the possible neuroprotective role of DHA may be explained by its capacity to restore normal brain *n*-3 PUFA metabolism (Bousquet et al., 2008; Lo Van et al., 2016). Accordingly, we next aimed to assess possible changes in fatty acid composition caused by administering TG-DHA in control and striatal 6-OHDA-lesioned mice. First, we examined the fatty acid profile in two brain areas, namely the striatum and cortex, of control and striatal 6-OHDA-lesioned mice. In control animals, saturated fatty acid content was significantly higher in the striatum, while unsaturated, monounsaturated, and polyunsaturated fatty acid content was higher in the cortex (**Table 1**). Thus, the amount of DHA in the striatum was significantly lower than in the cortex (**Table 1**), as previously reported (Létondor et al., 2014). In addition, the PI, the DBI, and the AI were significantly reduced in the striatum (**Table 1**). Since PI and DBI values could be related to vulnerability to peroxidative damage, the low levels of PI and DBI found in the striatum with respect to those observed in the frontal cortex may indicate that the striatum is especially sensitive to 6-OHDA-mediated neuronal damage, because these membranes are actively producing substrates for peroxidative modification. However, when evaluating the fatty acid profile of striatal 6-OHDA-lesioned animals, no differences were observed either in the striatum or the cortex (**Table 1**). In addition, a possible effect of the 6-OHDA lesion in the periphery, which could reflect some central effects, was also examined by determining the lipid content found in plasma and in total membrane extracts from RBC (**Table 1**). We found that the striatal 6-OHDA lesion significantly reduced PUFA and DHA levels in plasma of lesioned animals, but did not affect the lipid content of membrane extracts from erythrocytes (**Table 1**). Furthermore, PI and DBI were only significantly reduced in plasma of 6-OHDA-lesioned mice (**Table 1**). These differences might be related to greater 6-OHDA-mediated oxidation sensitivity of PUFA and DHA when associated with plasmatic proteins and lipoproteins (i.e., plasma) than when embedded in a biological membrane (i.e., RBC). Overall, the striatal 6-OHDA lesion had no effect on the fatty acid profile of the striatum, as recently reported (Coulombe et al., 2016), but did modify plasma fatty acid concentrations.

**Table 1:** Fatty acid composition of total lipids from plasma and total membrane extracts from red blood cell (RBC), cortex and striatum which were isolated from control (Ctrl) and 6-OHDA lesioned mice.

	% of Total fatty cids								
	
	Tissue:	Plasma		RBC		Cortex		Striatum	
					
	Mouse:	Ctrl	Lesioned	Ctrl	Lesioned	Ctrl	Lesioned	Ctrl	Lesioned
**Fatty Acid**:									
SFA		35 ± 0.5	35.3 ± 0.2	52.6 ± 2	53.3 ± 2.9	50.7 ± 0.4	49.9 ± 1.3	75.6 ± 1.7^¥¥¥^	73.3 ± 2.2
UFA		65 ± 0.5	64.7 ± 0.2	47.4 ± 2	46.7 ± 2.9	49.2 ± 0.4	50.1 ± 1.3	24.4 ± 1.7^¥¥¥^	26.7 ± 2.2
MUFA		16.3 ± 1.8	20.8 ± 1.2	18.3 ± 0.8	18.9 ± 1	24.1 ± 0.4	23.8 ± 0.2	18.2 ± 0.8^¥¥¥^	19.7 ± 0.3
PUFA		48.8 ± 1.3	↓ 44 ± 1.2^∗^	29.2 ± 2.1	27.8 ± 3.9	25.1 ± 0.6	26.3 ± 1.1	6.2 ± 1.1^¥¥¥^	7 ± 2
PUFA *n*-6		45 ± 1.3	41 ± 1.3	26.5 ± 1.7	25.1 ± 3.2	11.7 ± 0.4	12.3 ± 0.4	4 ± 0.7^¥¥¥^	4.4 ± 1.3
PUFA *n*-3		3.8 ± 0.09	↓ 3 ± 0.2^∗∗^	2.7 ± 0.3	2.7 ± 0.7	13.4 ± 0.3	14 ± 0.7	2.2 ± 0.4^¥¥¥^	2.5 ± 0.7
*n*-6/*n*-3 ratio		12 ± 0.6	13.6 ± 1	10.4 ± 0.9	11.7 ± 2.5	0.9 ± 0.02	0.9 ± 0.03	1.9 ± 0.1^¥¥¥^	1.9 ± 0.4
DHA		3 ± 0.1	↓ 2.2 ± 0.2^∗∗^	2.4 ± 0.3	2.4 ± 0.6	13.4 ± 0.3	14 ± 0.7	2.2 ± 0.4^¥¥¥^	2.5 ± 0.7
DHA + EPA		3.1 ± 0.1	↓ 2.3 ± 0.2^∗∗^	2.4 ± 0.3	2.4 ± 0.6	13.4 ± 0.3	14 ± 0.7	2.2 ± 0.4^¥¥¥^	2.5 ± 0.7
AA/DHA		4.6 ± 0.3	5.1 ± 0.4	5 ± 0.3	5.2 ± 0.7	0.6 ± 0.01	0.6 ± 0.02	1.6 ± 0.1^¥¥¥^	1.6 ± 0.3
PI		120.3 ± 3.6	↓ 101.3 ± 4^∗∗^	89.6 ± 9.2	86 ± 16.3	152 ± 3.3	158.9 ± 7.6	32.4 ± 6^¥¥¥^	37.6 ± 10.8
DBI		160 ± 2.5	↓ 145.8 ± 3^∗∗^	113.5 ± 8.1	109.5 ± 14.2	144 ± 1.9	148.8 ± 6	45.1 ± 5.4^¥¥¥^	50.1 ± 8.4
AI		36.7 ± 3.8	35 ± 2	30.1 ± 2.1	31.2 ± 2.2	162 ± 4.3	163 ± 5.4	67.5 ± 5.3^¥¥¥^	77 ± 17


*Data are presented as mean ± S.E.M. (*n* = 5 animals). ^∗^*P* < 0.05 and ^∗∗^*P* < 0.01 Student nest when compared to its control group. ^¥¥¥^*P <* 0.001 Student’s *t*-test when compared to cortex (control) group. SFA, saturated fatty acid; UFA, unsaturated fatty acid; MUFA, monounsaturated fatty acid; PUFA, polyunsaturated fatty acid; *n*-6, omega 6; *n*-3, omega 3; DHA, docosahexaenoic acid; EPA, eicosapentaenoic acid; AA, arachidonic acid; PI, peroxidizability index; DBI, double bond index; AI, anti-inflammatory index.*

**FIGURE 4:**
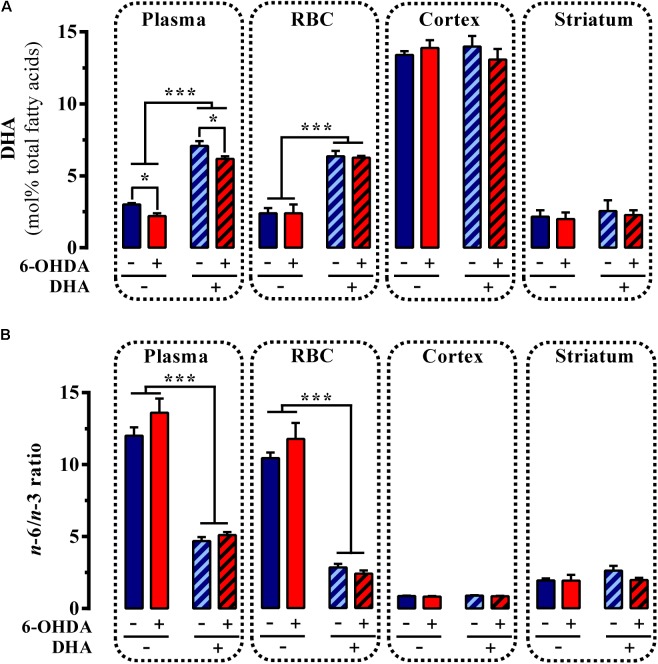
Effect of TG-DHA treatment on PUFA content. Control (Veh) and 6-OHDA-lesioned mice were administered with TG-DHA (250 mg/kg, i.g.) for 22 days before DHA concentration (molar %) was determined from plasma, RBC membranes, cortex, and striatum **(A)**. The PUFA *n*-6/*n*-3 ratio was also determined **(B)**. Data are presented as mean ± S.E.M. (*n* = 5 animals). ^∗^*P* < 0.05 and ^∗∗∗^*P* < 0.001 two-way ANOVA with Bonferroni’s *post hoc* test.

Subsequently, we assessed whether daily administration of the TG-DHA of DHA for 22 days (**Figure 2A**) had any impact on the PUFA profile of control and striatal 6-OHDA-lesioned animals. Interestingly, while an effect of 6-OHDA lesion [*F* 15.46, *P* < 0.01] and TG-DHA treatment [*F*_(1,16)_ = 344.4, *P* < 0.0001] on plasma DHA content was confirmed by two-way ANOVA analysis of the data, no effect of the interaction between both factors [*F*_(1,16)_ = 0.064, *P* = 0.803] was observed (**Figure 4A**). In addition, a significant reduction in plasma DHA content in 6-OHDA-lesioned animals, whether treated or untreated with TG-DHA (*P* < 0.05; **Figure 4A**), was confirmed by Bonferroni’s *post hoc* analysis. Similarly, while an effect of TG-DHA treatment [*F*_(1,16)_ = 94.5, *P* < 0.0001] on DHA content in RBC membranes was confirmed by two-way ANOVA analysis of the data, no effect of 6-OHDA lesion [*F*_(1,16)_ = 0.015, *P* = 0.902], or the interaction between both factors [*F*_(1,16)_ = 0.064, *P* = 0.803], was observed (**Figure 4A**). Neither the 6-OHDA lesion nor the TG-DHA treatment had any effect on DHA content in membranes from cortex and striatum (**Figure 4A**). Consequently, following chronic TG-DHA treatment, a significant reduction in the *n*-6/*n*-3 ratio was observed in plasma [*F*_(1,16)_ = 172, *P* < 0.0001] and RBC membranes [*F*_(1,16)_ = 193.9, *P* < 0.0001], but not in cortical or striatal membranes (**Figure 4B**). Overall, these results show that 22 days of TG-DHA administration prompted an increase in DHA content and a reduction in the *n*-6/*n*-3 ratio in plasma and RBC membranes, but did not alter the amount of DHA in membranes from cortex and striatum.

### TG-DHA Treatment Precluded 6-OHDA-Induced Locomotor Impairment in Mice

**FIGURE 5:**
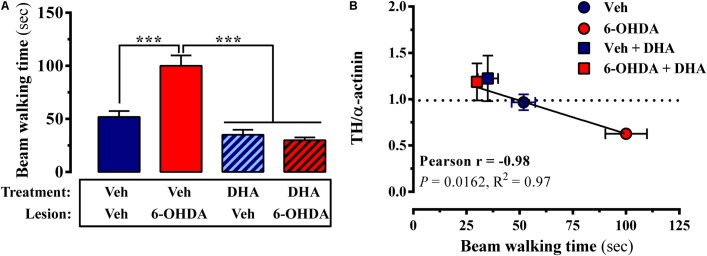
Impact of TG-DHA treatment on locomotor coordination. **(A)** Control (Veh) or 6-OHDA-lesioned mice, treated either with vehicle (Veh) or TG-DHA, were placed on the beam and the time taken to traverse was considered an indication of their motor function. Results are expressed as means ± SEM (*n* = 12 animals). ^∗∗∗^*P* < 0.001 two-way ANOVA with Bonferroni’s *post hoc* test. **(B)** Linear regression and correlation between TH expression and time taken (sec) to traverse the beam. Values are means ± S.E.M. (*n* = 12 animals).

According to the molecular data, 6-OHDA-lesioned mice chronically treated with TG-DHA should present an improved striatal function, since dopaminergic neurotransmission was less damaged. In order to assess a behavioral output related to possible alterations in motor skills, which are mainly controlled by the striatal basal ganglia, we used a very sensitive model to determine fine motor coordination and balance in mice and rats (Luong et al., 2011). As shown in **Figure 2A**, mice were subjected to the beam walking test, in which, after training, we measured the time taken to cross the beam (see methods). We observed that the 6-OHDA lesion significantly increased beam walking time, thus corroborating alterations in motor skills in this mouse model of PD (**Figure 5A**). However, 6-OHDA-lesioned mice that received long-term TG-DHA treatment exhibited similar beam walking times to wild-type mice, thus suggesting that TG-DHA exerted neuroprotective effects as regards behavioral function (**Figure 5A**). When TH expression and behavioral performance (i.e., beam walking time) were plotted, a significant inverse correlation was observed, yielding values of *R*^2^ × 0.97 (*P* < 0.05) and Pearson’s *r* = -0.98 (**Figure 5B**). Therefore, by assessing beam walking time we could, in theory, gauge the extent of dopaminergic innervation (i.e., TH expression). Overall, TG-DHA treatment attenuated 6-OHDA-toxic effects as assessed through TH expression and beam walking time (**Figure 5B**).

## Discussion

Parkinson’s disease is a neurodegenerative condition of unknown etiology, but plausible causes include oxidative stress and inflammation. Many drugs and nutrients have been postulated to prevent and/or palliate the processes associated with neurodegenerative diseases. Of these, *n*-3 PUFA such as DHA are among the most extensively studied. Here, we assessed the neuroprotective effect of TG-DHA in its TG-DHA using three different experimental models of parkinsonism: (i) primary neuronal cultures from striatum (*in vitro* model); (ii) striatal brain slices (*ex vivo* model); and (iii) 6-OHDA-lesioned animal model of PD (*in vivo* model). Our results support the notion that this molecule may represent a potential neuroprotective agent for PD.

First, we evaluated the *in vitro* and *ex vivo* effect of TG-DHA in primary striatal cultures and in striatal slices. As previously shown (Ding et al., 2004), treatment with 6- OHDA induced toxic effects in cells, which were partially blocked by TG-DHA. Thus, TG-DHA exhibited a modest but significant protective action *in vitro* and *ex vivo*. This observation is consistent with a previous study in which we showed that TG-DHA partially reversed induced cell death in striatal slices and primary striatal cultures (Fernández-Dueñas et al., 2017). Similarly, we recently observed that TG-DHA increased cell viability in BV-2 microglia cells activated with lipopolysaccharide and IFN-γ, although it only attenuated the production of some pro-inflammatory mediators (Mancera et al., 2017). A possible explanation for the relatively modest effects of TG-DHA-mediated protection against cell death *in vitro* might be that TG-DHA alters plasma membrane lipid turnover and/or fluidity differently *in vitro* vs. *in vivo*. When assessing DHA levels in control and 6-OHDA-lesioned mice, we did not observe an accumulation of DHA in brain tissue. Thus, we hypothesize that a dynamic turnover would better explain the effects of TG-DHA supplementation. Therefore, rather than producing an increase in fatty acid content, long-term administration of TG-DHA might counterbalance 6-OHDA-mediated oxidative damage and thus protect against disruption of normal brain PUFA metabolism (Bousquet et al., 2008). Meanwhile, a significant enrichment was found in plasma and RBC, which would be consistent with the view that blood lipid levels may act as a biomarker in neurodegenerative disorders (for review see Dyall, 2015). Of note, these it cannot be excluded that the peripheral changes could be secondary to the general state of the animals, rather than to the treatment.

Having ascertained the neuroprotective effects of TG-DHA in cell models, we studied an animal model, the 6-OHDA-lesioned mouse. We performed the 6-OHDA lesion in the striatum as it has been reported that striatal lesions lead to reliable, effective and retrograde degeneration of dopaminergic neurons within the substantia nigra pars compacta (Bagga et al., 2015; Becker et al., 2017), thus mimicking better the disease-associated temporal dopaminergic denervation (Deumens et al., 2002). Interestingly, we found that concomitant and subsequent chronic administration of TG-DHA completely prevented 6-OHDA-mediated striatal dopaminergic cell death; furthermore, treated mice exhibited similar motor skills to those of non-lesioned control mice. Various studies have investigated the neuroprotective effects of *n*-3 PUFA in animal models of PD. In contrast to our study, Coulombe and collaborators (Coulombe et al., 2016) recently found no improvements in motor behavior, but did observe partial neurorescue of the dopaminergic system in 6-OHDA-lesioned mice fed with a control or an *n*-3 PUFA-enriched diet for 6 weeks. However, their study differed from ours in several important respects: (i) Coulombe and collaborators performed a unilateral lesion in the right striatum, whereas we lesioned both striatal hemispheres; (ii) their treatment started 3 weeks after the lesion, whereas we administered TG-DHA immediately after 6-OHDA lesion; (iii) they evaluated dopaminergic damage in the substantia nigra pars compacta, whereas we assessed the lesion in striatum; and (iv) they used apomorphine-induced contralateral rotation as the behavioral output, whereas we evaluated motor coordination and balance through the beam walking test. Therefore, while both experimental approaches were suitable to assess parkinsonism in animal models of the disease, our approach differed in three main respects: (i) prophylactic TG-DHA administration; (ii) accurate measurement of motor skills; and (iii) evaluation of dopaminergic denervation by TH expression in the striatum. Therefore, our study provides complementary information to that previously reported by Coulombe and collaborators. In another study, Bousquet and collaborators reported that animals receiving prophylactic *n*-3 PUFA from 2 to 12 months were resistant to MPTP lesions, and no reduction in TH was observed (Bousquet et al., 2008). In a subsequent study, they assessed the relationship between brain-derived neurotrophic factor (BDNF) and the *n*-3 PUFA-mediated neuroprotective effect using the same animal model of parkinsonism. They found that *n*-3 PUFA pre-treatment for 10 months increased BDNF mRNA expression in the striatum, but not in the motor cortex of mice. Furthermore, *n*-3 PUFA treatment increased BDNF protein content in the motor cortex of parkinsonian animals. Overall, these results suggest that *n*- 3 PUFA modulation of BDNF expression may form part of the mechanism underlying *n*-3 PUFA-mediated neuroprotection in an animal model of parkinsonism (Bousquet et al., 2009). Conversely, in another study on rats subjected to chronic *n*-3 PUFA supplementation (over 90 days), it was observed that the 6-OHDA lesion induced a significant loss of TH expression, and again only a partial rescue of apomorphine-induced rotation was obtained (Delattre et al., 2010). Given the foregoing, we conclude that DHA consumption clearly correlates with partial or complete neuroprotection, but it may depend on the region examined; indeed, we observed large differences in the fatty acid profile when comparing cortex and striatum. While all previous studies have used behavioral tests that are not sufficiently sensitive to examine motor skills, here we implemented the beam walking test as a behavioral tool to assess DHA-mediated neuroprotection. This test has previously been shown to be useful to evaluate motor skills and balance in other neurodegenerative pathologies, such as Huntington’s disease (Luong et al., 2011), and we have now implemented it in a PD animal model.

In sum, we have shown the beneficial effects of administering TG-DHA on neural viability following 6-OHDA-induced toxicity, the tight control of brain lipid content even after dopaminergic denervation, and the utility of the beam walking test to robustly evaluate parkinsonism. We conclude that the use of TG-DHA as a neuroprotective agent in PD may constitute a promising pharmacotherapeutic strategy.

## Author Contributions

MG-S performed the biochemical and behavioral experiments. BC performed the lipid determinations. XM performed biochemical experiments VF-D conceived the experiments and wrote the paper. JD conceived the project and analyzed the results. FC conceived the project, analyzed the results and wrote the paper.

## Conflict of Interest Statement

The authors declare that the research was conducted in the absence of any commercial or financial relationships that could be construed as a potential conflict of interest.
